# Photoaging and theory-based intervention to improve sun protection behaviors in students in Zahedan: study protocol for a randomized controlled trial

**DOI:** 10.1186/s13063-023-07270-8

**Published:** 2023-06-26

**Authors:** Hassan Okati-Aliabad, Esmat Sadat Hosseini, Mohammad Ali Morowati Sharifabad, Mahdi Mohammadi, Mohamad Ebrahimzadeh Ardakani, Amir Hossein Talebrouhi

**Affiliations:** 1 Present Address: Health Promotion Research Center, Zahedan University of Medical Sciences, Zahedan, Iran; 2 PhD candidate of Health Education and Health Promotion,, Shahid Sadoughi University of Medical Sciences, Yazd, Iran; 3 Department of Health Education & Promotion, Shahid Sadoughi University of Medical Sciences, Yazd, Iran; 4 Department of Dermatology, Shahid Sadoughi University of Medical Sciences, Yazd, Iran; 5 Department of Computer Engineering, Imam Reza International University, Mashhad, Iran

**Keywords:** Face-aging interventions, Protection motivation theory, Ultraviolet, Sunshine, Mobile app

## Abstract

**Background:**

Excessive exposure to solar ultraviolet (UV) radiation can cause skin cancer. Implementing new technologies and computational algorithms can potentially change the outlook for cancer prevention and facilitate early detection of melanoma, thereby reducing mortality. Mobile technology as a potential provider of health services in delivering health information and conducting interventions, especially in skin fields, where a significant part of diagnosis is based on visual examination, can be important. Evidence showed that constructs of protection motivation theory (PMT) were good predictors of practicing sun protection behaviors in students. This study will investigate whether mobile applications improve safe and healthy behaviors and affect students' reduced UV exposure.

**Method/design:**

This randomized controlled trial will be conducted on 320 students on 06/04/2022 in Zahedan. We created mobile applications (Sunshine and Skin Health and WhatsApp apps). Sunshine and Skin Health app allows users to see their changed faces in three stages of adolescence, middle age, and old age based on sun protection behavior. The WhatsApp app has 27 health messages based on PMT theory, eight educational files, and a skin cancer clip that will be sent through WhatsApp during a week. Randomization will be performed using a 1:1 (control: intervention) ratio. The primary endpoint is the group difference in sun-protective behaviors and PMT constructs immediately after the intervention. The secondary endpoint is the group difference in sun-protective behaviors and PMT constructs at a 3-month follow-up. The data will be analyzed in SPSS.22, and the significance level will be considered at 0.05.

**Discussion:**

The present study examines the effectiveness of mobile applications in improving sun-protective behaviors. If this intervention enhances sun protection behaviors, it can prevent students’ skin damage.

**Trial registration:**

Iranian Registry of Clinical Trials IRCT20200924048825N1. Prospectively registered on 8 February 2021.

## Background and rationale

Sunlight exposure can be beneficial and harmful to health [1]. Long-term and continuous contact exposure to ultraviolet (UV) and recurrent sunburn, especially in the early years of life, increases the risk of skin cancer (especially melanoma and basal cell carcinoma), macular degeneration, and cataracts [2, 3]. Studies show that a significant proportion of skin cancer cases are due to excessive UV exposure [4, 5]. Skin cancer is one of the most serious public health problems and is the most common type of cancer in people with fair skin in many parts of the world [6]. Evidence from Iran has shown that due to high sunlight in most seasons and lack of proper coverage like outdoor clothing and hats, skin cancer has been increasing in recent years [7]. The annual UV index average map in Iran shows a very high index in the country’s southern half. The highest levels were observed in the southern states, especially in Sistan and Balochistan. This reflects the growing vulnerability of residents in these areas [8]. The most important foundation for preventing skin cancer is properly managing UV exposure to reduce or even prevent chronic sun damage, especially skin aging and skin cancer [9]. The following behaviors can reduce the damage caused by sunlight: shadow search, wearing appropriate sunscreen (including a wide-brimmed hat, matte UV sunglasses, and long-sleeve shirts), less exposure or no exposure to sunlight during peak hours (10 am to 4 pm), use sunscreen with SPF 30 or higher in areas without skin coverage, and avoid artificial sources of UV rays (fluorescent lamps, etc.) [10–12].

Over the last two decades, Mobile Health has provided health services exponentially through mobile phones and their applications [13]. In other words, healthcare providers use mobile technologies such as short message services (SMS) and mobile apps to provide health information and interventions [14]. Apps are software applications created to run a computer or mobile device to achieve a specific goal [15]; they can access remote databases, track time/place, and integrate user input to provide appropriate health information [16]. These apps are most important in skin care programs, where many diagnostics and follow-ups are based on visual examinations [17]. Implementing new technologies and computational algorithms can potentially change the outlook for cancer prevention and early detection of melanoma, consequently reducing the disease-specific mortality rate [18]. The increasing use of mobile phones is projected to reach more than six billion subscribers by 2021, which can be integrated into people’s daily lives to render health services to many people [19]. Today, the use of technology-based interventions is growing, such as the use of mobile applications [16], mails [20], and the use of SMS reminders (SMS text messages) for short message services [21, 22]. Facial aging interventions that change the user’s image and predict future appearance can motivate healthier behavior in the prevention of obesity, skin cancer, and smoking cessation. Preliminary results can be explained by the importance of the emergence of self-concept, especially in adolescence [23]. A systematic review of the interventions to promote sun protection behaviors in recreational settings showed poor and heterogeneous findings on the effectiveness of interventions. Consequently, it may be hypothesized that interventions over supportive social norms, which provide appearance-based information about skin aging photos (showing UV-damaged skin photos), are more effective than interventions on sun protection behaviors. Evidence indicates that using mobile technologies to promote health-related behaviors may be considered a new topic and a practical way to reach the target population [24]. Young people are increasingly moving away from traditional media such as newspapers and television and favoring web-based streaming. Web-based media has challenged the healthcare sector by creating new ways to reach young people with targeted personal advertising messages [25].

### Theory-based intervention to improve sun protection behavior

**Fig. 1:**
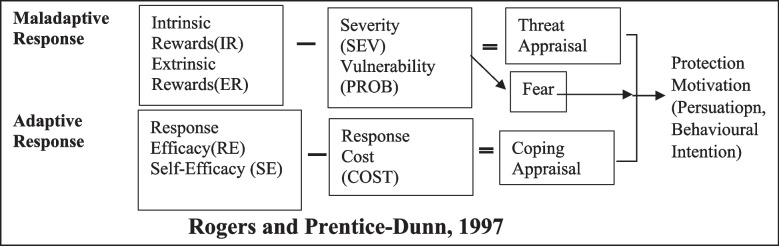
Constructs of PMT

Theories of social cognitive, planned behavior, and protection motivation in the field of behavior change provide useful insights to explain variables influencing the adoption of sun protection behaviors [23]. PMT was proposed by Rogers [26] based on the value expectation model to explain the effects of fear on health attitudes and behaviors. It offers a useful insight to explain the variables that affect the adoption of sun protection behaviors [27]. According to PMT, environmental and personal factors can pose a potential health threat which includes two cognitive processes: threat appraisal and coping appraisal. Threat appraisal examines factors influencing the likelihood of engagement in potentially unhealthy behaviors, including [1] perceived internal and external rewards of unhealthy behavior; [2] vulnerability: a person’s belief that one is vulnerable to a health threat; and [3] perceived severity: one’s belief that a health threat is serious. Fear is an intermediate variable between perceived vulnerability and perceived severity by assessing the threat. Coping appraisal evaluates the ability to deal with a threat and includes [1] perceived self-efficacy: a person’s belief that she can successfully perform the suggested behavior; [2] perceived response efficacy: a person’s assessment that the proposed behavior is effective; and [3] perceived cost: one’s estimate of the costs associated with performing the protective behavior. These two cognitive-mediated evaluation processes are combined to form the needed motivation for protection. Thus, adapting to a healthy behavior is a transient process of turning motivation into intention and behavior [28, 29]. Masoudi et al. reported that an educational intervention based on the theory of protective motivation was effective in promoting boys’ knowledge and behaviors in preventing UV damage [30]. Also, Ch’ng and Glendon [31] investigated the effectiveness of the PMT variables as predictors of sun protection behaviors in adolescents. They found a relationship between protection motivation and observed behavior, so adolescents paid particular attention to preventive measures to minimize sun exposure side effects (Fig. 1).

### Objectives

The overall objective of the trial is to determine the effectiveness of combining the Sunshine and Skin Health and WhatsApp apps intervention to improve sun-safe and healthy behaviors based on the protection motivation theory (PMT) among primary school students.

More specifically, the study aims are to compare the control and intervention groups over time (before (T1) and immediately after the intervention (T2) and at the 3-month follow-up (T3)):

1. Sun protection behavior.
2. PMT constructs including perceived vulnerability, perceived severity, response effectiveness, self-efficacy, reward, perceived response cost, fear, and protective motivation.

### Trial design

This randomized controlled trial (RCT) is an artificial intelligence technology-based education intervention with two-arm superiority parallel randomized controlled trial with a control group. The primary endpoint is the group difference in sun-protective behaviors and PMT constructs immediately after the intervention (T2). The secondary endpoint is the group difference in sun-protective behaviors and PMT constructs at 3-month follow-up (T3). Randomization will be performed using a 1:1 (control: intervention) ratio.

## Methods: participants, interventions, and outcomes

### Participants

Students in their fourth to sixth years of elementary school will be chosen as participants.

Inclusion criteria are as follows:

1. From age 10 to12 years old (from the fourth to sixth grade of elementary school).
2. Access to a smartphone, tablet, laptop, or computer.
3. Access to the Internet and social networks (e.g., WhatsApp)

Exclusion criteria are as follows:

1. Students who do not want to continue cooperation at any stage.
2. Illness or disorder making it impossible to perform the intervention program.

### Description of study settings

**Fig. 2:**
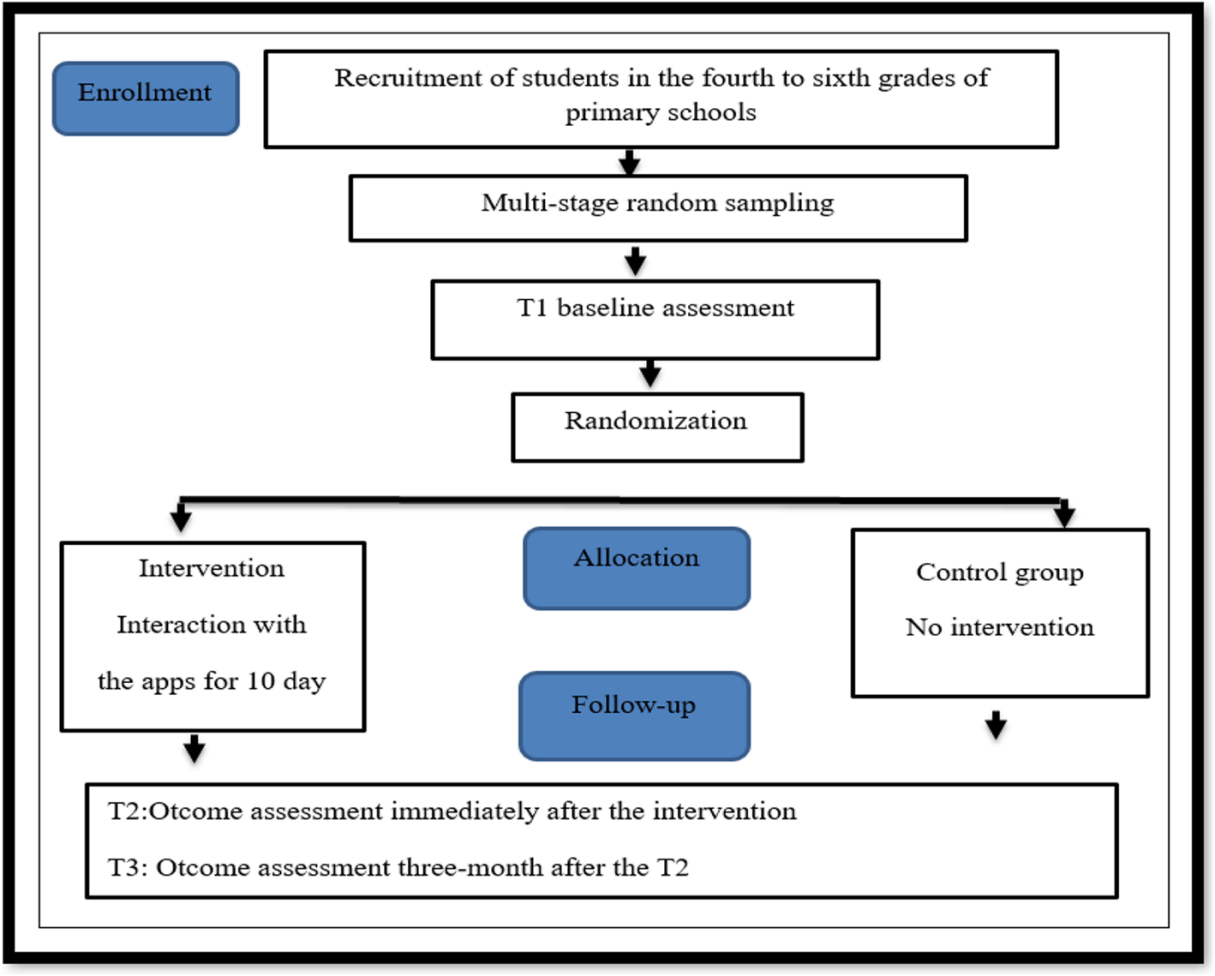
Trial diagram

This study is a randomized controlled trial (RCT) comparing intervention program with a control group in 10- to 12-year-old elementary school students. The project was implemented from April 2022 to July 2022. This period included participant recruitment, baseline data collection, and intervention (see Fig. 2). Randomization will be multi-stage random performed using a 1:1 (control: intervention) ratio. For this purpose, a list of elementary schools will be prepared. Thus, Zahedan city will be divided into five education districts and randomly selected four schools from each district (one girl’s and boy’s school as the intervention group and one girl’s and boy’s school as the control group). A self-administered questionnaire online will be provided to students through school information channels so that those who volunteer may participate in this research.

After approval of the Ethics Committee in Research of Shahid Sadoughi University of Medical Sciences (IR.SSU.SPH.REC.1399.135), a letter will be sent to the General Department of Education in Zahedan Province to obtain all elementary school lists and permission to conduct the research. The researcher will hold coordination meetings with school principals and explain the research clearly. During the COVID-19 prevalence, a specific application (the SHAD web) was developed by the ministry of education to be used for all schools for teaching, parental involvement, and other activities. Each school has this application. After the schools’ agreement, an announcement of the study will be posted on the schools’ channels. Online self-administered questionnaire will be provided to the target group through these school information channels (the SHAD web). Also, students’ written consent (electronically) is obtained before completing the questionnaire and documented in the experimental database. In addition to the consent of the students, the consent of the parents is also required. Thus, a link to an electronic consent form will be sent to parents. Parents who have agreed to the participation of their children send an electronic consent form to the researcher. After reviewing eligibility criteria, eligible students will complete baseline measures and then will be randomly divided into two groups (control and intervention) as detailed above and below.

### Randomization

The simple randomization will be done to assign participants for study groups by an independent statistician with a 1:1 allocation. A list of schools will be provided by referring to the Department of Education. Thus, Zahedan city will be divided into five education districts and randomly selected four schools from each district (one girl’s and boy’s school as the intervention group and one girl’s and boy’s school as the control group). A total of at least 20 elementary schools in Zahedan, with approximately 320 participants, will participate by simple randomization methodology. Once the students have consented and completed the baseline assessments, they will be randomized to intervention and control groups. From each school, the students selected will receive a special code through SPSS software, and after analyzing the data, they will be selected randomly through these codes. The independent statistician who has done the randomization informs the students by phone. Due to the nature of the intervention, it is impossible to blind participants and interventionists during the study. Still, outcome assessors will be blinded to group allocation in the final database as new identification numbers are assigned to participants. We will use the waiting-list control group design. After assessment T3, the control group will participate in the intervention program. This will be done so that both groups can experience the program. If participants withdrew or discontinued the educational intervention, researchers made efforts to encourage them to continue the study through two contacts. Also up to three text messages will be sent as reminders to complete questionnaires. Non-responders will be followed up by phone during the valuation period. Additionally, to increase adherence, digital notifications and reminders were sent to participants reminding them to visit and use the health messages, educational files, and skin cancer clip.

### Procedure

In order to implement the intervention, a free WhatsApp messaging program under the title of Sunshine and Skin Health will be developed. In this program, the researcher will introduce the study and the researchers; the purpose of the study and the rules of participation in the study will be explained to the participants. Also, the execution process of the plan will be explained in detail. They will receive an invitation to participate in the intervention. Completing the questionnaires immediately after the intervention(T2) and 3-month follow-up (T3) will be done in this program.

### Interventions

#### Sunshine and Skin Health app

Considering the comprehensive access of students to mobile phones and also the interest of adolescence in their appearance, we decided to design an app that allows the user to take a selfie and identify skin color, eye color, gender, and age based on the photo. In the next step, the user must answer seven behavioral questions [32] on a 5-point Likert scale ranging from 1 “never” to 5 “always” as described below:

1. How often did you use sunscreen last month?
2. When the intense sun, you are engaged in outdoor activities (fun, games, shopping, etc.), how often do you get in the shade?
3. How often do you wear a wide-brimmed sun hat when exposed to exposure sunlight during peak hours (10 am to 4 pm)?
4. How often do you wear long-sleeved clothing when exposed to the harsh sun?
5. How often do you wear sunglasses when exposed to sunlight during peak hours (10 am to 4 pm)?
6. How often do you use sunscreen when exposed to sunlight during peak hours (10 am to 4 pm)?
7. Do you use sunscreen again after washing your hands and face?

**Fig. 3:**
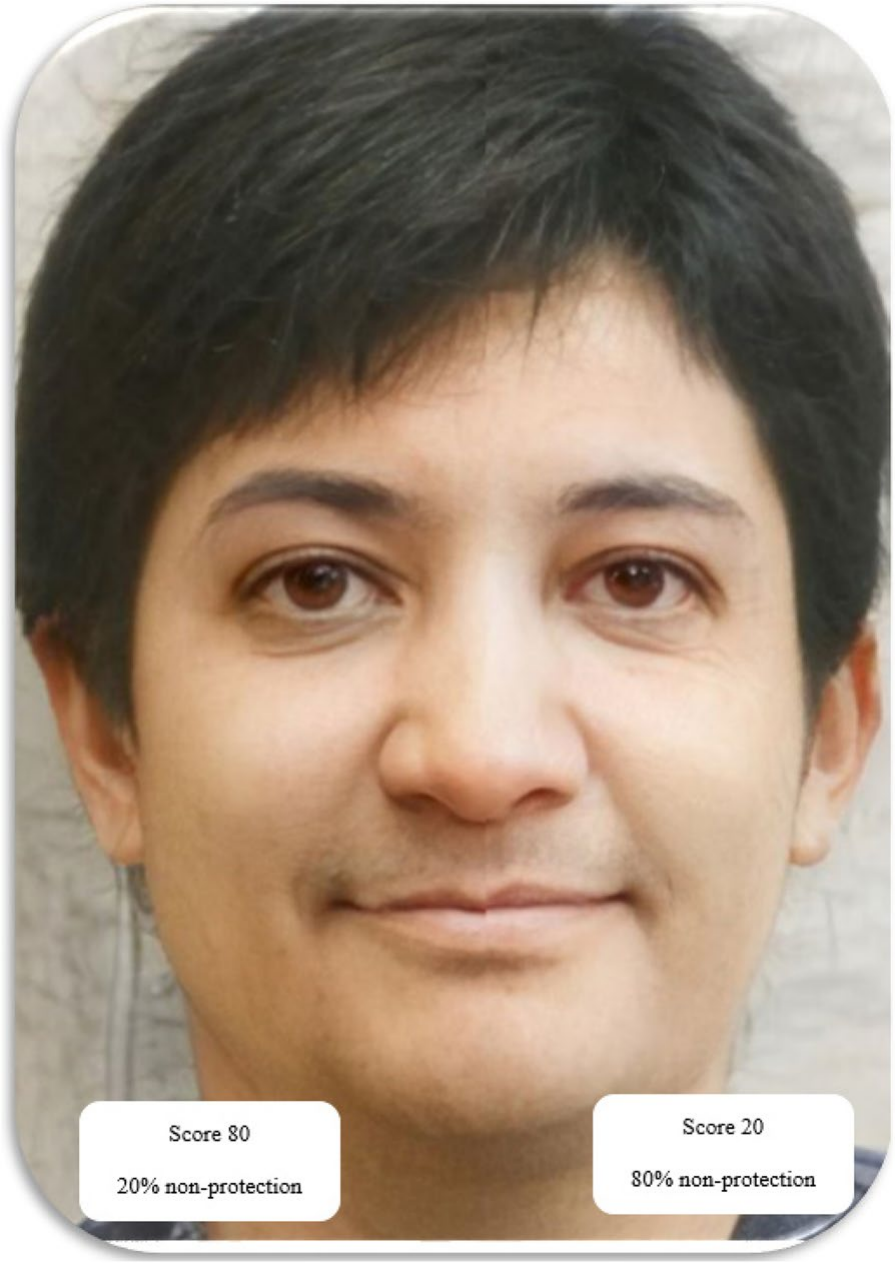
Graphics of program implementation in the adolescence phase

**Fig. 4:**
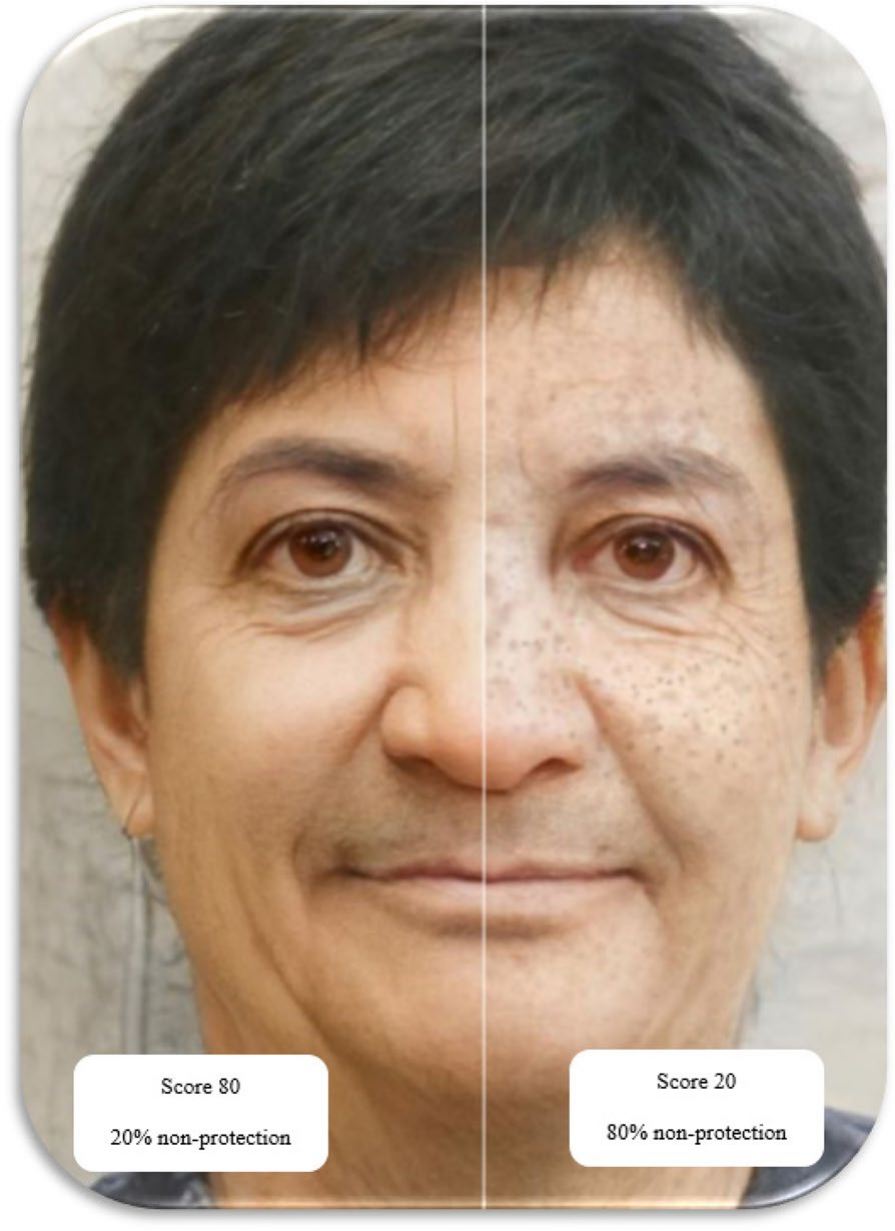
Graphics of program implementation in the middle age phase

**Fig. 5:**
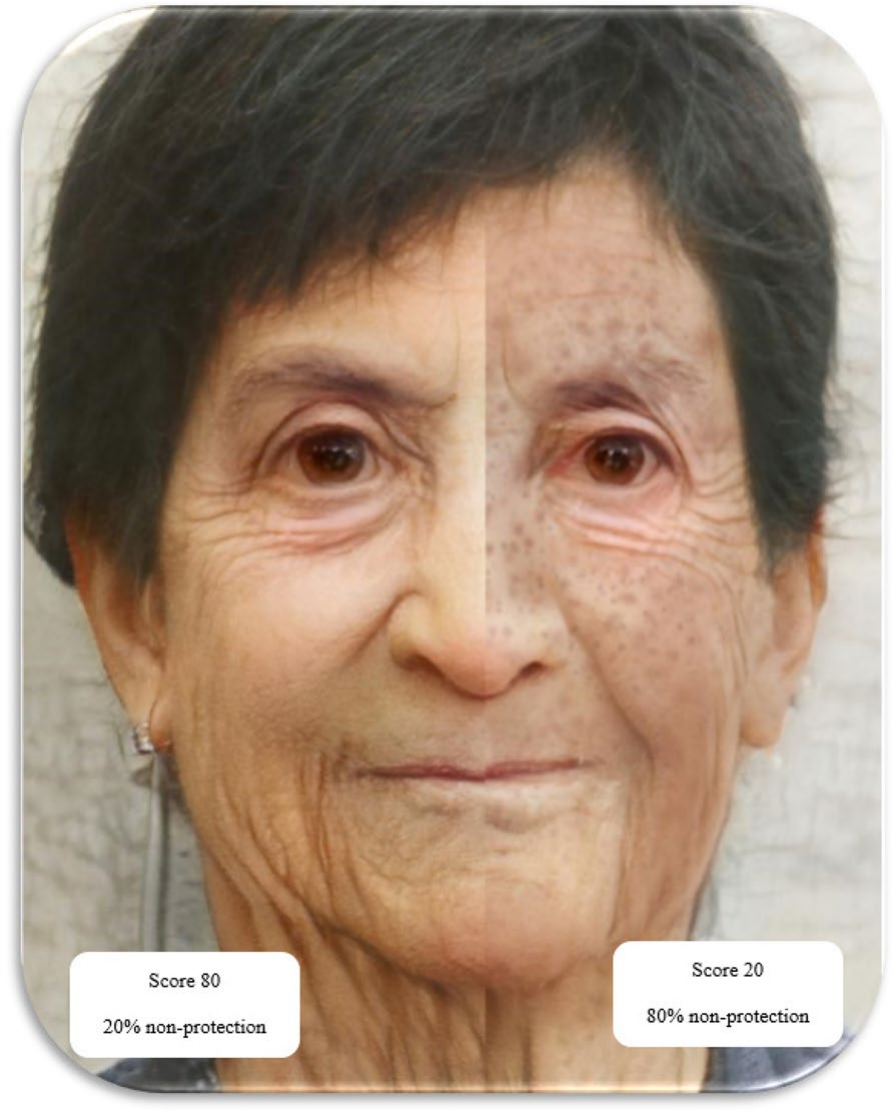
Graphics of program implementation in the aging phase

In the end, the student will see his changed face in the three stages of adolescence, middle age, and old age. Then, educational messages and recommendations about skin cancer risk and prevention methods will be displayed based on the user’s score (Figs. 3, 4, and 5).

The app also has the following capabilities:

1. It can be run on all platforms.
2. This app also offers messages and educational recommendations appropriate to each person’s skin color and eye color.
3. In addition to the app itself detecting skin color, eye color, gender, and age, the user can also enter data manually.

#### WhatsApp app

A 1-week cooperation invitation message is displayed at the end of working with the Sunshine and Skin Health app. The intervention includes 27 health messages based on PMT theory, eight educational files, and a skin cancer clip sent through the WhatsApp app during a week (10 am to 4 pm). At the end of each day, a question related to the health messages, educational file, or clip is proposed, and the participant sends his answer in audio or text. Messages are based on the scientific literature on topics related to improving sun protection behavior [7, 12, 33]. The educational content will be prepared after an extensive literature review on sun protection interventions [33–39]. The educational content will include using sunscreen, looking for shadows, wearing a brimmed hat and sunglasses, and wearing long-sleeved clothes. A video clip will be made with the content on skin importance, symptoms, diagnosis and treatment of skin cancer, change of moles, and ways to prevent skin.

### Strategies to improve adherence to interventions

The app has an attractive design and is easy to use, which helps improve adherence to interventions.

### Relevant concomitant care permitted or prohibited during the trial

There will be no restrictions on concomitant care, which will be monitored carefully during the trial through the questionnaire.

### Outcomes

**Fig. 6:**
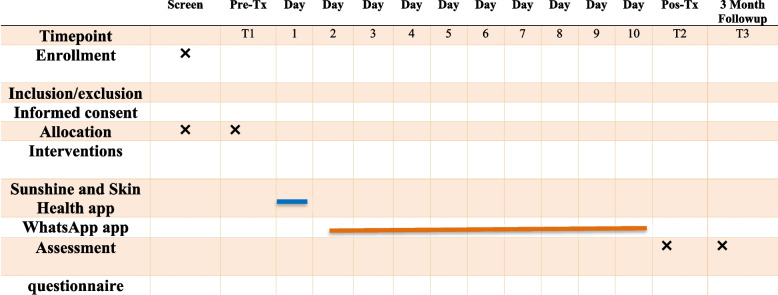
Study design schedule following the Standard Protocol Items Recommended for Clinical Trials (SPIRIT) guidelines

Data collection from demographic information to measuring sun protection behavior, and PMT constructs including perceived vulnerability, perceived severity, response effectiveness, self-efficacy, reward, perceived response cost, fear, and protective motivation in students will be done with an online self-administered questionnaire. Figure 6 provides a SPIRIT depiction of the schedule of enrollment, interventions, and assessments**.**

#### Primary outcome

The primary outcome of this study is the resultant changes between the two groups (intervention and control) immediately after the intervention (T2) in parameters including sun protection behavior and PMT constructs including perceived vulnerability, perceived severity, response effectiveness, self-efficacy, reward, perceived response cost, fear, and protective motivation.

#### Secondary outcomes

The secondary outcome of this study is the resultant changes between the two groups (intervention and control) at the 3-month follow-up (T3) in parameters including sun protection behavior and PMT constructs including perceived vulnerability, perceived severity, response effectiveness, self-efficacy, reward, perceived response cost, fear, and protective motivation.

### Questionnaires

Data will be collected through a self-administered questionnaire at three points: (T1) pre-randomized baseline, (T2) immediately after the intervention, and (T3) 3-month follow-up. All participants will complete a questionnaire that includes socio-demographic characteristics, sunscreen behavior, and PMT constructs.

Socio-demographic characteristics include age, gender, educational level, and parents’ jobs.

Sun protection behaviors will be measured through 7 questions: three questions about using sunscreen and four questions about other protective behaviors, including wide-brimmed sun hat, sunglasses, clothing, and shade-seeking.

The PMT constructs include susceptibility, severity, response efficacy, self-efficacy, reward, perceived response cost, fear, and protection motivation.

### Sample size

The sample size is calculated to detect a minimum relative difference of 20% between both groups at post-assessment. This effect was based on the previous studies’ primary outcome and mean protection motivation scores. Based on the relevant literature [30], with alpha error at 0.05 and statistical power at 0.80, and protection motivation before mean ± SD (5.39 ± 2.17) and after mean ± SD (7.01 ± 1.75), ∆ = 2, *ρ* = 0.05, and *n* = 20 was estimated as 160 people. Therefore the total sample size is 320.

### Data storage and management

All documentation, including informed consent and enrollment information booklets, will be stored with the principal investigator. No personal information will be recorded in the researcher’s WhatsApp contact, and to maintain confidentiality, all student personal information will be removed and replaced with a password-protected RID. After the end of the study period, the data will be stored on a protected computer. They are stored with a password. Only people who are part of the research team will have access to the data. The Steering Committee will hold online meetings every 6 months.

### Data analysis

Descriptive statistics will be used for socio-demographic characteristics including age, gender, educational level, and parents’ jobs. Data will be analyzed with linear mixed models taking into account the repeated measures design of the study. All assessment points will be included in the analyses. The main effect of time and group and the interaction between time and group will be assessed. The primary outcome of this study is the resultant changes between the two groups (intervention and control) immediately after the intervention (T2) in parameters including sun protection behavior and PMT constructs. The secondary outcome of this study is the resultant changes between the two groups (intervention and control) at the 3-month follow-up (T3). Estimates of mean between-group changes from T1 to T2 and T2 to T3 will also be provided. For primary outcomes, an alpha level of 0.05 will be applied. Additional analyses including subgroup analyses will be conducted, for example, to compare the effect of the intervention on behavior and the constructs of protection motivation theory in both boys and girls. *T*-test analyses will be used for between-group mean comparisons for normally distributed continuous data. For sensitivity analysis, all demographic variables will be compared between two groups before data analysis, and if there is a variable that has a significant difference between the two groups, it will be explored in analyses. Also for the behavior variable, we think that the participants’ answers to some behavioral questions will be different, so the behavior variable will be divided into several parts. The primary analysis will be based on the intervention intention population and therefore includes data from all participants providing baseline data previously randomly assigned to one of the two study conditions. Multiple imputation methods will be used to estimate missing data. We plan to perform all statistical analyses following intention to intervention model. Questionnaires are available for participants through a link sent in WhatsApp. The questionnaire is designed in a way that every participant must complete all of the questions to be allowed to send the questionnaire back. If an individual does not send the questionnaire back, we will text a recall message 3 times. If we still do not receive the questionnaire, we will call them. Therefore, we do not expect a large proportion of missing values. If there are, we may use multiple imputation methods to estimate missing data. In case of missing value existence, we may also compare the two groups in every evaluation time after intervention (the day after intervention, 3 months after intervention) in addition to using a repeated measures model.

### Auditing

In order to review and verify the development of the study throughout the trial period, the Steering Committee will hold online meetings every 6 months.

### Protocol amendments

If necessary, decisions on protocol amendment will be made by the research team and authorized by the ethics committee.

## Discussion

This study is an intervention based on artificial intelligence technology. New technologies based on artificial intelligence effectively use accepted theories of behavior change in health [40–42]. To the best of our knowledge, this study is the first in Iran to use artificial intelligence technology based on motivation theory to create a mobile application. This mobile application allows users to see their changed faces in three stages of adolescence, middle age, and old age based on sun protection behavior. If successful, the results show that the Sunshine and Skin Health app intervention and WhatsApp app intervention can promote sun-safe behaviors. Studies have shown that the risk of skin cancer is primarily associated with early sun exposure; risk groups are often unconscious. This study provides an opportunity to evaluate innovative and scalable appearance-based interventions in high-risk populations.

### Limitations

The academic recruitment will officially start in October 2021. However, due to Iran’s policies (stay at home) and (half-attendance of schools) due to the COVID-19 pandemic, we were concerned about low sun exposure during the pandemic. So for probable biases, we have decided to postpone the primary outcome data collection to the spring of 2022 and the intervention will be performed after the data collection.

### Ethics and dissemination

Iranian Registry of Clinical Trials (IRCT) has approved the study protocol (approval number: IRCT20200924048825N1) according to the Standard Protocol Items: Recommendations for Interventional Trials (SPIRIT) [43]. The results will be published in prestigious journals and conferences.

### Ethical consideration

Throughout the process, all participants will be assured of their anonymity.

## Trial status

Protocol version 3.0, protocol date February 8, 2021. Recruitment opened in September 2022 and will continue until all students required for the trial are enrolled, planned to be in March 2022. The study period will be 1 year long and will be finished in February 2022.

## Data Availability

The datasets generated and/or analyzed during the current study are not publicly available due to ongoing study but are available from the corresponding author upon reasonable request.

## Appendix

Consent form

## Abbreviations

PMT: Protection motivation theory
UV: Ultraviolet
SMS: Short message services
